# R&D investment and total factor productivity in China’s pharmaceutical industry: The moderating effect of the centralized procurement policy

**DOI:** 10.1371/journal.pone.0324519

**Published:** 2025-05-19

**Authors:** Xiujuan Li, Jiachun Xu

**Affiliations:** 1 Economics and Management School, Wuhan University, Wuhan, China,; 2 School of Economics, Jinan University, Guangzhou, China; Loughborough University, UNITED KINGDOM OF GREAT BRITAIN AND NORTHERN IRELAND

## Abstract

We use the implementation of China’s centralized procurement policy as a quasi-natural experiment to examine the effects of China’s centralized procurement policy on pharmaceutical research and development (R&D) investment and total factor productivity (TFP). Empirical findings demonstrate that the positive impact of R&D investment on TFP has a delayed effect in the pharmaceutical sector. The centralized procurement policy shortens this lagged effect by imposing survival pressure and pushing companies to enhance their R&D investment intensity significantly. From the heterogeneity perspective, for companies facing significant financing constraints, the centralized procurement policy significantly enhances the intensity of R&D investment. In contrast, for companies with weaker finance limitations, the impact of the policy on their R&D investment intensity is insignificant. Still, the positive moderating effect of the policy on the relationship between R&D investment and TFP is more pronounced. Based on the findings, policymakers need to take a long-term view and consider the diversified influence of different enterprises. Policies that reduce financing constraints and create an enabling environment for sustained R&D are important and complementary.

## 1. Introduction

Research and Development (R&D) investment is an essential component of technological advancement, significantly enhancing total factor productivity (TFP) [1]. Which not only reflects the quality of economic growth but also encompasses factors such as technological progress, management efficiency, and institutional innovation [2]. Existing research indicates that the positive impact of R&D investment on TFP often emerges after a certain lag period. The pharmaceutical industry highly depends on innovation and R&D [3], and Pharmaceutical R&D activities are characterized by significant investment, long cycles, and high risks. In the pharmaceutical industry, the impact of R&D investment on total factor productivity (TFP) exhibits a unique dynamic relationship. Research on U.S. pharmaceutical industry has found that the impact of R&D investment on productivity begins to manifest 3–5 years later and continues to strengthen in the following years [4]. The lag effects indicate that pharmaceutical companies should have a long-term vision when setting up R&D strategies, and also raise higher demands for the policymakers when formulating industrial policies.

Since 2018, the Chinese government implemented a national centralized drug procurement policy. The government procures certain drugs through a uniform bidding process and guarantees the quantity of procurement to the winning enterprises. The pharmaceutical companies competed by quoting prices; the bidder who presented the lowest price would win the contract and gain a significant market share. Through the national centralized procurement policy, the government aims to reduce drug prices and lower the burden of medical insurance while also establishing standards for generic drugs [5]. The national centralized procurement policy in China has already had a profound impact on the Chinese pharmaceutical industry, lowering drug prices and alleviating patients’ financial burdens [6]. The policy has also significantly altered the competitive landscape of the pharmaceutical market and has profound effects on pharmaceutical companies’ R&D strategies and innovation behavior [7]. On the one hand, the significantly reduced drug prices have compressed the profit margins of companies, thereby affecting the internal funds available for research and development expenditure; on the other hand, the survival pressure has compelled companies to intensify their innovation efforts to sustain a competitive advantage and shift towards the development of high-value-added innovative drugs and high-end generics [8].

This study employs the quasi-natural experimental of the national centralized procurement policy to examine its impact on pharmaceutical businesses’ R&D expenditure and the intricate relationship among policy, R&D investment, and total factor productivity (TFP). Specifically, we focus on three core questions:

The lagged effect of R&D on TFP: Through empirical analysis, we explore how R&D investment impacts TFP over different periods and reveal the specific manifestation of this dynamic relationship.

Influence of policy on R&D investment: To analyze the promotional effect of the centralized Procurement Policy on R&D investment in Pharmaceutical Companies, we examine the changes in R&D investment before and after the policy implementation to explore how the policy alters companies’ innovation strategies.

The Synergistic Effect of Policy and R&D Investment on TFP: With the centralized procurement policy as a moderating variable for R&D investment, we analyze its impact on the mechanism through which R&D investment influences total factor productivity (TFP).

This paper unfolds as follows: in Section 2, we examine pertinent literature and formulate our hypotheses. Section 3 elucidates our methodology and describes the research design, including data, variables, and our models. Section 4 presents the empirical results, including robustness checks and an in-depth discussion. Finally, we conclude with theoretical implications and policy optimization direction.

## 2. Literature review and research hypotheses

### 2.1. R&D investment and total factor productivity

The correlation between R&D investment and TFP is a fundamental subject in the fields of economics and innovation management study. Solow (1957) first highlighted the contribution of technological advancement in the growth of economy [9]; extensive theoretical and empirical studies have examined how R&D expenditure affects TFP. Early theoretical work laid the foundation for understanding this relationship. Cohen and Levinthal (1989) proposed the idea of “absorptive capacity,” emphasizing that R&D efforts enable enterprises to enhance their productivity while also absorbing external knowledge and technology, leading to additional productivity gains [10]. Romer (1990), in his endogenous growth model, illustrated the essential function of R&D in propelling economic development through increasing production efficiency via knowledge accumulation, thereby boosting TFP [11].

Subsequent empirical studies demonstrate a robust positive association between R&D investment and TFP while also revealing heterogeneity across industries. Acemoglu et al. (2006) found that R&D investment in high-tech sectors significantly influences productivity improvement [12]. In a later study, Acemoglu et al. (2018) observed similarly strong productivity effects of R&D in technologically advanced and developing industries, suggesting that the impact of R&D differs across industries [13]. Doraszelski and Jaumandreu (2013) demonstrated that the returns to R&D vary significantly across firms, even within the same industry, underscoring the importance of firm-specific factors [14]. Lucking et al. (2019) updated estimates of R&D spillovers and discovered that the societal returns on R&D remain substantial and have not diminished over time, addressing apprehensions of diminishing returns[15]. Boeing and Mueller (2019), examining R&D–productivity dynamics in China, found that private firms experience significant productivity gains from R&D, while state-owned enterprises do not [16]. Mohnen and Hall (2013) reviewed the innovation–productivity literature and emphasized the need to consider different types of innovation (product, process, organization) when assessing R&D’s impact [17]. Cirera and Maloney (2017) emphasized that complementary factors such as human capital and management practices are crucial for assessing the efficacy of R&D investment, particularly in developing countries [18]. Corrado et al. (2022) examined the increasing significance of intangible capital and digital technologies, highlighting that these trends are transforming the character of R&D and its relationship with productivity [19].

As the study advanced, experts discovered that R&D investment influences TFP in neither an instantaneous nor linear way but instead exhibits significant time-lag effects. Griffith et al. (2004) and Van Reenen (2011) provided early evidence of this lag, showing that R&D efforts often take several years to translate into productivity gains, particularly in high-technology sectors [20,21]. Hall et al. (2010) further confirmed that the positive impact of a firm’s R&D investment on productivity growth typically requires several years to manifest [1]. Bloom et al. (2020) found that during the initial phase of R&D investment, firms might experience a short-term decline in productivity [22]. This phenomenon could be due to resource reallocation, curve effects, and integrating new technologies with existing production systems. Their research indicates that the impact of R&D investment on TFP exhibits a “J-curve” effect: There might be negative effects initially, but these effects transform into significant positive ones over time.

In light of the above study of how R&D investment affects TFP through various mechanisms and time frames, we put forth the subsequent hypothesis:

Hypothesis 1: R&D investment can improve the total factor productivity of pharmaceutical companies, but there is a lag effect.

### 2.2. Impacts of the centralized procurement policy

The centralized procurement policy has significantly influenced the pharmaceutical industry since its implementation in 2018. Researchers have studied the impacts of this policy on different aspects of the industry, including the change of drug prices, corporate financial performance, R&D innovation, and industry structure.

Regarding drug prices, multiple studies consistently show that the centralized procurement policy has dramatically reduced drug bidding prices. Lin et al. (2022), analyzing the first batch of centralized procurement, found that bid prices dropped by an average of 72.2%, procurement volumes increased by 16.9%, and procurement costs fell by 55.3% [23]. Sun et al. (2023) further noted that these price reductions alleviated patients’ financial burdens and curtailed excessive profit margins in the pharmaceutical market [24]. However, Chen and Zhang (2020) raised concerns about potential declines in drug quality, suggesting that some smaller pharmaceutical firms might lower quality standards to cut costs under intense price pressure [25].

In terms of corporate financial performance, the policy’s impact is also significant. Hua et al. (2022) reported that the revenues and profit margins of winning firms initially declined after the policy’s introduction, but gradually recovered as those firms’ market shares expanded and production costs decreased [26]. By contrast, firms that did not win bids experienced substantial downturns in financial performance due to lost market share. Additionally, Hu et al. (2021), using an event study on stock prices around policy announcements, found that winning companies’ stock prices showed significant short-term volatility but eventually recovered as the market adjusted to the new policy [8].

Regarding R&D and innovation, several researchers argue that the centralized procurement policy compels companies to strengthen their innovation initiatives. Huang and Tao (2020) noted that to deal with fierce pricing competition, the policy compels pharmaceutical companies to enhance research and development efficiency and innovation capability, which potentially promotes industrial upgrading in the long run [27]. Mo (2022) also argued that the procurement policy accelerates industry-wide competitiveness improvements, thereby driving innovation [28]. Empirically, Cheng (2023) applied a difference-in-differences model and found that the centralized procurement policy significantly enhanced firms’ innovation, which secured bids [29]. These studies suggest that although the policy lowers profit margins in the short term, it can incentivize companies to shift toward higher-value drug innovation and promote long-term advancement of the industry.

Furthermore, the policy’s influence on the industry structure has drawn attention. Li et al. (2024) found that, while significantly lowering drug prices, the procurement policy also incentivized firms to increase investment in innovative drug R&D [30]. Li and Xu (2024) noted that by squeezing the profit margins of generic drugs, the policy prompted a strategic shift toward high-value innovative drug development, which could increase industry concentration and promote higher-quality growth in the pharmaceutical sector [7]. However, the implementation of the policy also faces challenges. Zhou et al. (2024) observed that although the policy provides firms with more stable market expectations and reduces uncertainty in R&D expenditure, it simultaneously increases financing pressures on companies [31]. Maintaining R&D investment under the policy’s price pressures has become a critical issue to small and medium-sized enterprises.

This study proposes the following hypothesis, according to the aforementioned analysis and regarding the influence of centralized procurement policies on pharmaceutical companies’ R&D expenditure decisions.

Hypothesis 2: The centralized procurement policy approach substantially enhances R&D expenditure by pharmaceutical firms.

### 2.3. The centralize procurement policy, TFP, and R&D investment

The relationship between government policy, corporate R&D investment, and TFP a prominent topic in economics and innovation research. With the rise of innovation-driven development strategies, scholars have increasingly examined how these three elements interact.

The influence of government policy on R&D investment has been extensively examined. David et al. (2000) conducted a thorough assessment and discovered that government R&D subsidies markedly enhance firms’ R&D investment, especially amplifying innovation efforts in small and medium-sized enterprises (SMEs) [32]. Similarly, Czarnitzki and Hussinger (2004) showed that tax incentives effectively reduce firms’ innovation costs, enhancing their propensity to invest in R&D activities [33]. Yu et al. (2016) discovered that supportive industrial policies significantly enhance corporate innovation activities in China, Particularly in private businesses [34].

Researchers have also investigated the direct influence of government policies on total factor productivity(TFP). Aghion et al. (2015) demonstrated that industrial and technology policies can enhance TFP by optimizing resource allocation and promoting technology diffusion [35]. Hanushek and Woessmann (2015) emphasized that education and training policies indirectly boost productivity by improving workforce quality and skill levels [36]. However, Zhang et al. (2019) pointed out that certain industrial policies may adversely affect the TFP of targeted sectors, although a well-developed market environment can mitigate these adverse effects [37].

Recent research has increasingly concentrated on the moderating influence of policy in the correlation between R&D investment and TFP. Qian et al. (2018) discovered that industrial policy would improve capital allocation efficiency and enhance companies’ total factor productivity [38]. Foreman-Peck and Zhou (2022) analyzed public R&D subsidies in Eastern European countries and discovered that government subsidies positively affect TFP, particularly for smaller firms [39]. Similarly, Li et al. (2022) illustrated that government subsidies in China alleviate enterprises’ financial constraints and promote productivity growth via innovation [40].

The institutional environment significantly influences supporting innovation and productivity improvement. Qiu et al. (2021) analyzed the relationship among institutional quality, R&D investment, and TFP in China, highlighting the importance of strong intellectual property protection and healthy market competition in fostering innovation and productivity growth [41]. Matt et al. (2022) emphasized that to translate R&D investment into productivity improvements, a well-functioning innovation ecosystem—including supportive policies and institutions—is essential [42]. However, the policy influenced the relationship between R&D investment and TFP in a heterogeneous manner. Barajas et al. (2021) researched how public policies in Spain influence business productivity and R&D investment. According to their findings, the efficacy of these policies varied depending on firm characteristics, such as size and innovation intensity [43]. As Hu et al. (2022) noted, government subsidies impact on firm’sproductivity in China is also complex. For instance, state-owned enterprises obtain more substantial benefits than private firms [44].This study proposes the subsequent hypothesis based on the aforementioned analysis of the potential moderating effect of the centralized procurement policy on the correlation between R&D expenditure and total factor productivity(TFP).

Hypothesis 3: The relationship between R&D investment and total factor productivity (TFP) is substantially moderated by the centralized procurement policy.

#### 2.4. Financing constraints, total factor productivity (TFP), and R&D investment.

The relationship between R&D investment and TFP may vary depending on the financial constraints faced by pharmaceutical firms; access to capital is a vital component of the innovation process, and this links back to the moderating role of the centralized procurement policy. Financial sources are more diversified in companies with lower financing constraints. And they can seek external funding through (e.g., equity or debt), rather than relying on solely on internal cash flow (Brown et al., 2009) [45]. Under the market pressure resulting from the procurement policy, these enterprises can quickly and flexibly adjust their R&D strategies and increase R&D investment more rapidly and flexibly adjust their R&D strategies and raise R&D investments (Czarnitzki & Toole, 2011) [46]. They can use external financing to support new R&D projects or increase investments in existing projects without overly depending on operating cash flow. This flexibility allows them to adjust to policy changes more efficiently, speed up the R&D process, and thus mitigate the negative effect of R&D investment on total factor productivity (TFP) in the preliminary period, taking positive effects sooner.

In contrast, companies with stronger financing constraints face greater challenges. These firms predominantly depend on internal cash flow to finance their R&D endeavors, rendering R&D investment more vulnerable to short-term business conditions. Following the enactment of the procurement policy, if a company’s short-term profits are negatively impacted, it may lead to cutbacks or delays in R&D investment. Research by Himmelberg and Petersen (1994) indicates that for financially constrained companies, R&D expenditure have a strong correlation with internal cash flow [47]. Although the procurement policy creates incentives to increase R&D investment, these companies might struggle to raise or maintain their R&D spending. The procurement policy’s positive moderating influence on the correlation between R&D investment and total factor productivity (TFP) for enterprises facing significant constraints would be weakened as a result.

We propose the subsequent hypothesis based on the above analysis of how financing constraints could affect the policy’s moderating role.

Hypothesis 4: Firms that are afflicted by weaker financing constraints exhibit a more substantial positive moderating influence of the procurement policy on the R&D–TFP relationship.

## 3. Research methodology

### 3.1. Sample and data sources

The initial cohort for this study consisted of publicly 416 listed pharmaceutical companies that were classified under the pharmaceutical and biotechnology sector (as per the 2014 Shenwan Industry Classification Classification).

Following the exclusion of firms with incomplete data and those designated under Special Treatment (ST) status due to financial distress or other issues, the final sample consists of 163 pharmaceutical companies, comprising 1,467 annual observations. To comprehensively examine the effects of the centralized procurement policy, our sample period spans from 2015 to 2023, covering nine batches of procurement under the policy.

We manually compiled a directory of listed pharmaceutical companies and their subsidiaries, and identified those participating in the by referencing the national list of drugs under the centralized procurement policy (sourced from the Shanghai Sunshine Procurement website). This step confirmed each company’s inclusion in the procurement scope. Firm-level financial data and additional company attributes were acquired from the China Stock Market & Accounting Research (CSMAR) database.

### 3.2. Variables

#### 3.2.1. Total factor productivity.

This study employs TFP as the dependent variable. Traditional production function estimation may suffer from endogeneity issues where input factors (e.g., capital and labor) correlate with productivity shocks (e.g., technological innovations), a phenomenon known as simultaneity bias that can lead to biased parameter estimates (Marschak & Andrews, 1944). To address this methodological challenge, we employ the semiparametric estimation approach presented by Levinsohn and Petrin (2003) [48] (hereafter LP method), which utilizes intermediate inputs (e.g., raw materials and energy consumption) as proxy variables to capture unobservable productivity shocks. Specifically, the LP method assumes the production function is:


$$y_{t}=\beta_{0}+\beta_{l}l_{t}+\beta_{k}k_{t}+\beta_{m}m_{t}+\omega_{t}+\varepsilon_{t}$$ (1)

Where $y_{t}$ represents total output, $l_{t}$ represents labor input, $k_{t}$ represents capital input, $m_{t}$ represents intermediate input, $\omega_{t}$ represents productivity shocks, and $\varepsilon_{t}$ represents random errors.

Estimation of $\omega_{t}$ is carried out in two stages:

Initial Stage Estimation: Utilize intermediate inputs $m_{t}$ as a surrogate for productivity shocks $\omega_{t}$. Estimate the coefficient of labor $\beta_{l}$ using non-parametric methods such as polynomial regression.

Second Stage Estimation: Utilize the results from the first stage to construct moment conditions. Generalized Method of Moments (GMM) is applied to estimate the coefficients for capital $\beta_{k}$ and intermediate inputs $\beta_{m}$.

Finally, obtain a consistent and efficient estimate of $\omega_{t}$ based on the estimated parameters and observed inputs.

Consistent with production function literature, we operationalize key variables as follows:

1. Total Output $y_{t}$: Following Bond et al. (2003) [49], we proxy firm-level output using inflation-adjusted revenue, addressing potential measurement biases in physical output metrics common to multi-product firms.
2. Labor Input $l_{t}$: Building on He and Yang (2012) [50], we measure labor input through total cash compensation (wages, benefits, and training expenditures) rather than headcounts. This approach captures Skill heterogeneity via wage differentials, Human capital investments (e.g., training programs), and Temporal intensity adjustments (overtime/seasonal payments).
3. Capital input $k_{t}$: assessed with the net value of fixed assets [51].
4. Intermediate Inputs $m_{t}$: Computed as the sum of the firm’s operating costs, administrative expenses, financial expenses, and sales expenses, minus the depreciation and amortization for the period, along with the cash payments to employees and for employee-related costs.

#### 3.2.2. Centralized procurement policy.

This study examines the drug centralized procurement policy, which is extensive and encompasses all nine batches since its establishment in 2018. This is represented by a binary variable, denoted as CP. ${CP}_{t}={Treat}_{i}\times {Post}_{t}$, where ${Treat}_{i}$ equals 1 if the drugs produced by firm i are within the procurement scope, and 0 otherwise; ${Post}_{t}$ equals to 1 for the year t and subsequent years if the drugs produced by the firm are included in the procurement batch of year t, and 0 otherwise.

#### 3.2.3. R&D investment.

In line with Li and Yu (2015)), we use R&D intensity as the measure of a company’s R&D investment [52]. R&D intensity is characterized as the proportion of a company’s research and development expenditure relative to its revenue. This relative measure controls for differences in company size, allowing for a direct comparison of innovation investment across firms of different sizes.

#### 3.2.4. Financial constraints.

We employ the SA index as an indicator for the financing restrictions encountered by each company, following the methodology established by Hadlock and Pierce (2010) [53] and Yu et al. (2019) [54].The KZ and WW index was once predominant; however, the SA index provides a more straightforward and resilient assessment of financing constraints. A elevated SA index number signifies that a company encounters more significant funding limitations in its operations.

#### 3.2.5. Control variables.

Based on previous research [52,54], we incorporate the subsequent control variables in our regressions to account for other factors that may affect TFP or R&D:

• Firm Size (SIZE): The natural logarithm of total assets at the end of the year.
• Return on Equity (ROE): Net profit divided by the average shareholders’ equity (net assets) for the year (expressed as a percentage).
• Leverage Ratio (LEV): Average total liabilities divided by average total assets for the year (expressed as a percentage).
• Government Subsidies (SUBSIDY): The natural logarithm of the monetary value of government subsidies received during the year.
• Ownership Concentration (OC): The ratio of shares owned by the largest shareholder relative to the total shares owned by the top ten shareholders (a measure of ownership structure).

Table 1 presents the information on the variables used in this study.

**Table 1:** Main variables information.

Variable Name	Variable Symbol	Variable Definition
Total Factor Productivity	TFP	Estimated using the Levinsohn-Petrin method.
Procurement Policy	CP	If the company’s drug, which has passed consistency evaluation, is included in the procurement range for year t, then centralized procurement is 1 for year t and onwards; otherwise, it is 0.
R&D Investment	RD	The proportion of R&D investment relative to total revenue.
Financing Constraints	SA	Measured using the SA index from Hadlock and Pierce (2010).
Firm Size	SIZE	The natural logarithm of total assets at the end of the year.
Return on Equity (%)	ROE	Net profit divided by average net assets.
Leverage Ratio (%)	LEV	Average total liabilities divided by average total assets for the year.
Government Subsidies	SUBSIDY	Government subsidies received in the current year.
Ownership Concentration (%)	OC	The proportion of shares held by the largest shareholder relative to the total shares held by the top ten shareholders.

### 3.3. Model specification

To examine the lagged effects of R&D investment on TFP, this study uses R&D investment for the current period and lags of 1–5 periods as explanatory variables. We formulate the subsequent fixed effects model to analyze the influence of R&D investment on TFP:


$${TFP}_{it}=\alpha +\beta {RD}_{it-j}+\delta X_{it}+\gamma_{i}+v_{t}+\varepsilon_{it}\;(j=0,1,2,3,4,5)$$ (2)

${TFP}_{it}\;$: Total factor productivity of company i in time t.

${RD}_{it-j}(j=0,1,2,3,4,5)$: represents the R&D investment of company I and its lagged values.

$X_{it}$: the control variables.

$\gamma_{i}$: firm fixed effects.

$v_{t}$: time fixed effects.

$\varepsilon_{it}$: the random error term.

Hypothesis 2 is tested by comparing R&D investment between firms affected by the policy (treatment group) and those not affected (control group) prior to and after policy implementation with a difference-in-differences (DID) approach. Model estimation is conducted as follows:


$${RD}_{it}=\alpha +\beta {CP}_{it}+\delta X_{it}+\gamma_{i}+v_{t}+\varepsilon_{it}$$ (3)

Where ${RD}_{it}$ is the R&D investment intensity, ${CP}_{it}$ is a binary variable for centralized procurement policy. $X_{it}$ are the control variables.

To study hypothesis 3, how the centralized procurement policy affects R&D investment and total factor productivity of pharmaceutical enterprises. The model can be written as:


$${TFP}_{it}=\alpha +\beta_{1}{RD}_{it}++\beta_{2}{CP}_{it}+\beta_{3}{RD}_{it}*{CP}_{it}+\delta X_{it}+\gamma_{i}+v_{t}+\varepsilon_{it}$$ (4)

Where ${RD}_{it}*{VBP}_{it}$ is the interaction term between centralized procurement policy and pharmaceutical R&D investment.

### 3.4. Descriptive statistics

Summary statistics for the primary variables are presented in Table 2. The mean TFP among sample firms is 8.215, with values ranging from 5.029 to 11.679. The mean R&D intensity (RD) is approximately 6.392%, with a minimum of 0.008% and a maximum of 74.33%. This suggests that there is a significant degree of variability in R&D investment among firms.

**Table 2:** The primary variables’ descriptive statistics.

	Obs.	Mean	Std. Dev.	Min	Median	Max
**TFP**	1,467	8.215	0.964	5.029	8.114	11.679
**RD**	1,467	6.392	6.082	0.008	4.840	74.330
**VBP**	1,467	0.168	0.374	0.000	0.000	1.000
**SA**	1,467	-3.934	0.230	-4.838	-3.920	-3.270
**SIZE**	1,467	84.184	157.557	1.228	37.857	2119.725
**ROE**	1,467	8.309	28.007	-488.984	9.774	460.738
**LEV**	1,467	33.654	17.219	2.550	31.599	114.493
**SUBSIDY**	1,467	6.483	18.334	0.000	0.201	93.945
**OC**	1,467	48.456	23.734	0.001	50.677	97.652

## 4. Empirical results

### 4.1. Benchmark test

#### 4.1.1. R&D investment and TFP.

Table 3 reports the impact of R&D investment on TFP (corresponding to Model 2). Columns (1)–(6) represent the estimated effects of current R&D and R&D lagged by one to five years, respectively.

**Table 3:** R&D investment and total factor productivity.

	(1)	(2)	(3)	(4)	(5)	(6)
	**TFP**	**TFP**	**TFP**	**TFP**	**TFP**	**TFP**
**RD**	-0.021***					
	(0.002)					
**RD (-1)**		-0.019***				
		(0.002)				
**RD (-2)**			-0.009***			
			(0.002)			
**RD (-3)**				0.001		
				(0.003)		
**RD (-4)**					0.002	
					(0.003)	
**RD (-5)**						0.007**
						(0.003)
**Constant**	7.021***	6.810***	6.436***	6.141***	6.095***	6.348***
	(0.091)	(0.102)	(0.114)	(0.132)	(0.150)	(0.194)
**Control variables**						
**Company dummies**	1,467	1,304	1,141	978	815	652
**Year dummies**	0.948	0.954	0.961	0.967	0.973	0.980
**Observations**	7.021***	6.810***	6.436***	6.141***	6.095***	6.348***
**R-squared**	(0.091)	(0.102)	(0.114)	(0.132)	(0.150)	(0.194)

Note: All tests are controlled for individual and time-fixed effects as well as characteristic variables of the enterprise. This article omits the regression findings of the control variables due to spatial constraints. The parentheses indicate robust standard errors, *** p < 0.01, ** p < 0.05, * p < 0.1.

The results from columns (1) through (6) reveal that current-period R&D investment has a considerable negative effect on TFP, and this negative impact persists during the first and second lag years. From the third lag year onward, the negative influence reduces and eventually turns positive. By the sixth year, R&D investment significantly increases the TFP of pharmaceutical companies, supporting Hypothesis 1.

These findings illustrate the dynamic nature of R&D’s contribution to productivity. In the short term, diverting resources to research and development can reduce current operational efficiency and output, as R&D projects require substantial funds, personnel, and time and do not yield immediate returns. However, after a period of accumulation and maturation, R&D outcomes start to translate into actual productivity benefits through new or improved products, the implementation of new technology, streamlined production processes, or innovations in management practices. These outcomes enhance production efficiency, lower costs, improve product quality, and create new market opportunities, thereby ultimately increasing TFP.

#### 4.1.2. Centralized procurement policy and R&D investment.

As outlined in Model (1), Table 4 illustrates the influence of the centralized procurement policy on R&D investment. Column (1) presents the double fixed effects estimation results without control variables, while Column (2) shows results with control factors incorporated. At the 1% significance level, the centralized procurement policy variable (CP) is substantially positive, suggesting that the procurement policy significantly enhances R&D investment intensity in pharmaceutical enterprises.

**Table 4:** Centralized procurement policy and R&D investment.

	(1)	(2)
	**RD**	**RD**
**CP**	1.230***	1.103***
	(0.408)	(0.406)
**Constant**	6.184***	7.338***
	(0.107)	(1.706)
**Control variables**	No	Yes
**Company dummies**	Yes	Yes
**Year dummies**	Yes	Yes
**Observations**	1,467	1,467
**R-squared**	0.695	0.703

Note: All tests are controlled for individual and time-fixed effects. Due to space limitations, this article did not report regression results for control variables. The parenthesis denotes robust standard errors, *** p < 0.01, ** p < 0.05, * p < 0.1.

Pharmaceutical companies are encouraged to increase their R&D investment in innovative drugs, first generics, and high-end generics as a result of the policy, which lowers drug prices and compresses profits for low-end generic drugs. This policy not only drives technological advancement and structural upgrading in the pharmaceutical industry but also provides patients with more high-quality and affordable drug options, supporting Hypothesis 2.

#### 4.1.3. Centralized procurement policy, R&D investment, and TFP.

The results of the moderating influence of the CP policy on the connection between R&D and TFP in Model (3) are presented in Table 5. Column (1) displays the results of the double fixed effects estimation without control variables, while Column (2) includes control variables. The interaction term RD*CP has a positive and statistically significant coefficient (at the 5% significance level), demonstrating that the drug centralized procurement policy significantly strengthens the relationship between R&D investment and TFP in pharmaceutical companies, consistent with Hypothesis 3.

**Table 5:** Centralized procurement policy, R&D investment, and TFP.

	(1)	(2)
	TFP	TFP
RD	-0.032***	-0.027***
	(0.003)	(0.002)
CP	-0.164***	-0.065*
	(0.039)	(0.034)
RD*CP	0.009***	0.006**
	(0.003)	(0.003)
Constant	8.436***	6.999***
	(0.019)	(0.097)
Control variables	No	Yes
Company dummies	Yes	Yes
Year dummies	Yes	Yes
Observations	1,467	1,467
R-squared	0.923	0.944

Note: All tests are controlled for individual and time-fixed effects. This article did not report the regression results for control variables due to space limitations. The parentheses indicate robust standard errors, *** p < 0.01, ** p < 0.05, * p < 0.1.

First, the drug centralized procurement policy provides firms with a more stable and predictable market demand (through guaranteed large purchase volumes for winning bidders), which reduces uncertainty and encourages companies to commit resources to R&D with greater confidence in future returns. Second, the procurement process intensifies price competition via bidding, prompting firms to enhance product quality and reduce production costs through R&D in order to win contracts and stand out in the competitive market. This heightened competitive pressure boosts companies’ innovation motivation, prompting them to concentrate more on market-oriented R&D and increase their R&D innovation efforts’ efficiency. Third, the policy may induce a reallocation of resources within the industry, allowing these firms to receive more resources, so enhancing the overall R&D performance and productivity. This indicates that the drug centralized procurement policy directly influences R&D investment. At the same time, it also alters the way R&D investment impacts total factor productivity.

### 4.2. Robustness test

To verify the robustness of our outcomes—particularly the drug centralized procurement policy’s positive moderating influence on the R&D–TFP relationship, we employed alternative measures. We recalculated the firm’s TFP with four different methods: OLS, fixed effects estimation, Olley-Pakes, and GMM, replacement of the LP method. Table 6 summarizes the outcomes of these robustness checks. The interaction term RD * CP remains positive and statistically significant at roughly the 5% level across all methodologies. This consistency indicates a positive moderating effect of the relationship between R&D investment and total factor productivity (TFP). This validates the robustness of the findings displayed in Table 5.

**Table 6:** Robustness test results.

	(1)	(2)	(3)	(4)
	TFP_OLS	TFP_FE	TFP_OP	TFP_GMM
RD	-0.026***	-0.025***	-0.028***	-0.029***
	(0.002)	(0.002)	(0.002)	(0.002)
CP	-0.081**	-0.086**	-0.045	-0.039
	(0.033)	(0.034)	(0.031)	(0.033)
RD*CP	0.008***	0.008***	0.007***	0.006**
	(0.003)	(0.003)	(0.003)	(0.003)
Constant	8.798***	9.341***	5.693***	4.831***
	(0.095)	(0.097)	(0.090)	(0.094)
Control variables	Yes	Yes	Yes	Yes
Company dummies	Yes	Yes	Yes	Yes
Year dummies	Yes	Yes	Yes	Yes
Observations	1,467	1,467	1,467	1,467
R-squared	0.959	0.962	0.921	0.906

Note: All tests are controlled for individual and time-fixed effects. This article did not report regression results for control variables due to space limitations. The parentheses denote robust standard errors, *** p < 0.01, ** p < 0.05, * p < 0.1.

### 4.3. Mechanism test

The centralized procurement policy’s impact on R&D investment in pharmaceutical firms with varied levels of finance limitations is illustrated in Table 7. Column (1) provides the fixed effects results regarding the influence of the procurement policy on finance limitations, Column (2) displays the findings for pharmaceutical companies with high finance limitations and Column (3) shows the results for those with low finance limitations.

**Table 7:** The influence of centralized procurement on R&D investment under different financing constraints.

	(1)	(2)	(3)
	SA	R&D Investment	
		Strong	Weak
CP	0.012***	1.672**	0.115
	(0.004)	(0.692)	(0.446)
Constant	-3.844***	5.851***	12.053***
	(0.016)	(2.156)	(2.159)
Control variables	Yes	Yes	Yes
Company dummies	Yes	Yes	Yes
Year dummies	Yes	Yes	Yes
Observations	1,467	651	798
R-squared	0.978	0.731	0.825

Note: All tests are controlled for individual and time-fixed effects. This article did not report regression results for control variables due to space limitations,. The parentheses indicate robust standard errors, *** p < 0.01, ** p < 0.05, * p < 0.1.

Column (1) displays a regression coefficient that is significantly positive at the 1% level for the centralized procurement policy’s influence on finance limitations. This suggests that the procurement policy increases financing constraints for pharmaceutical companies. The policy lowers drug prices, which significantly impacts drug manufacturers’ profit margins. When profits decline and internal cash flow decreases can lead to external investors and financial institutions’ pessimism about the company’s future profitability, which in turn increases the difficulty and expense of financing. This process intensified financing constraints, making it difficult for these companies to obtain necessary funds at reasonable costs.

In Columns (2) and (3), in pharmaceutical companies with substantial finance limitations, the centralized procurement policy considerably promotes R&D investment. However, the effect is not noticeable for companies with low finance limitations. To enterprises with high finance limitations, the policy forces them to seek new growth areas and competitive advantages under cost pressures, making R&D investment a critical avenue for overcoming challenges and boosting product competitiveness. Conversely, enterprises with low finance limitations due to better financial conditions and more available resources already possess strong R&D capabilities and competitive advantages, so the policy’s direct incentive effect is relatively small.

Table 8 illustrates the moderating effect of the centralized procurement policy on R&D investment and TFP under varied finance limitations. The findings of the double fixed-effect test on the moderating effect of the centralized policy on R&D investment and TFP under strong finance limitations are presented in Column (1), while the results under weak financing constraints are presented in Column (2).

**Table 8:** The moderating effect of centralized procurement under different financing constraints.

	(1)	(2)
	TFP	
	Strong	Weak
RD	-0.017***	-0.038***
	(0.004)	(0.004)
CP	-0.202**	-0.099**
	(0.079)	(0.048)
RD*CP	0.001	0.010**
	(0.006)	(0.005)
Constant	8.244***	8.722***
	(0.109)	(0.091)
Control variables	Yes	Yes
Company dummies	Yes	Yes
Year dummies	Yes	Yes
Observations	651	798
R-squared	0.955	0.954

Note: All tests are controlled for individual and time-fixed effects. Due to space limitations, regression results for control variables were not reported in this article. The parentheses indicate robust standard errors, *** p < 0.01, ** p < 0.05, * p < 0.1.

The centralized procurement policy has a substantially positive moderating effect on pharmaceutical companies with weak financing limitations, as evidenced by the regression coefficients of the interaction term RD*CP. Conversely, this positive moderating effect is not significant for companies with rigorous financing constraints. Due to the difficulty in obtaining funds for companies with strong financing constraints, even if they increase R&D investment under the pressure of the CP policy, their overall resources remain insufficient, resulting in insignificant improvements in TFP. Conversely, companies with weak financing constraints have relatively ample funds and resources to reinforce their R&D activities. Under the pressure of the CP policy, they can more effectively translate increased R&D investment into productivity improvements, thereby significantly enhancing TFP.

## 5. Discussion

### 5.1. Comparison with existing literature

Research on China’s centralized procurement policy mainly focuses on its impact on drug prices and social welfare, and mainly with case study analysis. Our study empirically extends research from the impact of China’s centralized procurement system on corporate R&D investment, as well as the influence of the policy on the relationship between R&D investment and TFP. Additionally, through heterogeneity analysis, our study examined the impact and function mechanisms of centralized procurement policies on R&D investment and TFP in enterprises with different financing constraints. This enriches the empirical research on China’s centralized procurement policy.

The relationship between R&D investment and TFP has been studied by numerous scholars from the characteristics of different industries in various countries. This paper, from the perspective of China’s pharmaceutical industry, investigates the relationship between R&D investment and TFP, enriching the existing research in terms of country and industry practices.

### 5.2. Future research directions

Future research can delve into the response of different kinds of companies (e.g., large firms vs. small and medium-sized firms) to the centralized procurement policy and how these differences interact with financial constraints, and influence R&D strategies and productivity improvements. Additionally, cross-national comparative studies could provide a broader evaluation of policy effectiveness, offering valuable benchmarks for policy optimization. Through these in-depth studies, we can provide stronger theoretical backing and policy direction for the sustainable development of the pharmaceutical industry.

## 6. Conclusions

Since 2018, China has implemented nine rounds of the centralized procurement in pharmaceutical industry. The government effectively consolidated medical institutions’ procurement needs, and enhanced its bargaining power with pharmaceutical manufacturers. The policy has increased competition in the pharmaceutical sales sector and enhanced transparency in the procurement process. Although the policy imposes greater survival pressure on pharmaceutical companies, it also compels them to intensify their focus on R&D innovation and improve production efficiency to maintain their competitive edge in an increasingly cost-conscious market.

This study reveals the relationship among China’s centralized procurement, R&D investment, and TFP, providing new perspectives and empirical evidence for evaluating and formulating industrial policies. The findings offer significant policy insights for the government on how to effectively promote innovation in the pharmaceutical industry while control medical costs. Specifically, our findings can assist policymakers in assessing the short-term impact of medical cost control and long-term innovation incentives in the pharmaceutical industry, providing a theoretical basis and data support for subsequent policy optimization. It also guides pharmaceutical firms in formulating effective R&D strategies under the policy.

The findings also reveal important policy implications for innovation and productivity improvement in China’s pharmaceutical sector. With the changing relationship between R&D spending and productivity, the effect of increased innovation may not be realized immediately. So it is important to have a stable, predictable policy environment — one that is conducive to sustained R&D effort over extended periods of time. These can include complementary actions such as enhancing the protection of intellectual property, granting targeted R&D subsidies or tax benefits, and engaging in human capital formation to set the stage for sustainable innovation in the long run. Moreover, these results highlight the importance of studying how policies behave heterogeneously across different types of firms. Policymakers should pay more attention to small and medium-sized enterprises (SMEs) and firms without significant external financial resources. Targeted policies to ease financing constraints—such as improving access to credit, creating capital markets for innovative firms, or offering government-backed loan guarantees—may create a more level playing field so a broader array of firms can partake in innovation-led growth.

## Supporting information

S1 Dataset Dataset. (CSV)
